# Expression Landscape and Functional Roles of HOXA4 and HOXA5 in Lung Adenocarcinoma

**DOI:** 10.7150/ijms.70445

**Published:** 2022-03-06

**Authors:** Li Gao, Rong-Quan He, Zhi-Guang Huang, Guo-Sheng Li, Jiang-Hui Zeng, Jia-Yin Hou, Jia-Yuan Luo, Yi-Wu Dang, Hua-Fu Zhou, Jin-Liang Kong, Da-Ping Yang, Zhen-Bo Feng, Gang Chen

**Affiliations:** 1 Department of Pathology, The First Affiliated Hospital of Guangxi Medical University, No.6 Shuangyong Rd, Nanning, Guangxi Zhuang Autonomous Region, 530021, P.R. China.; 2 Department of Medical Oncology, The First Affiliated Hospital of Guangxi Medical University, No.6 Shuangyong Rd, Nanning, Guangxi Zhuang Autonomous Region, 530021, P.R. China.; 3 Department of Cardio-Thoracic Surgery, The First Affiliated Hospital of Guangxi Medical University, No.6 Shuangyong Rd, Nanning, Guangxi Zhuang Autonomous Region, 530021, P.R. China.; 4 Department of Clinical Laboratory, The Third Affiliated Hospital of Guangxi Medical University/Nanning Second People's Hospital, No. 13 Dancun Road, Nanning, Guangxi Zhuang Autonomous Region, 530031, P. R. China.; 5 Department of Pathology, The Second Affiliated Hospital of Nanjing Medical University, No.121 of Jiangjiayuan, Nanjing, Jiangsu Province, 210000, P.R. China.; 6 Ward of Pulmonary and Critical Care Medicine, Department of Respiratory Medicine, The First Affiliated Hospital of Guangxi Medical University, No. 6, Shuangyong Road, Nanning, Guangxi Zhuang Autonomous Region, 530021, P.R. China.; 7 Department of Pathology, Guigang People's Hospital of Guangxi/The Eighth Affiliated Hospital of Guangxi Medical University, No. 1, Zhongshan Middle Road, Guigang, Guangxi Zhuang Autonomous Region, 530021, P.R. China.

**Keywords:** HOXA4 and HOXA5, *in vitro* experiments, lung adenocarcinoma, microarray, RNA-seq

## Abstract

Background: The role of HOXA family genes in the occurrence and progression of a variety of human cancers has been scatteredly reported. However, there is no systematic study on the differential expression, prognostic significance and potential molecular mechanism of HOXA4 and HOXA5 in LUAD.

Methods: In-house immunohistochemistry (IHC), multi-center microarrays, RT-qPCR and RNA-seq data were incorporated for comprehensively evaluating the expression and prognostic value of HOXA4 and HOXA5 in LUAD. The mechanism of HOXA4 and HOXA5 in the formation and development of LUAD was analyzed from multiple aspects of immune correlations, upstream transcriptional regulation, functional states of single cells and co-expressed gene network. The functional roles of HOXA4 and HOXA5 in LUAD were validated by *in vitro* experiments.

Results: As a result, in 3201 LUAD samples and 2494 non-cancer lung samples, HOXA4 and HOXA5 were significantly downexpressed (P < 0.05). The aberrant expression of HOXA5 was significantly correlated with the clinical progression of LUAD (P < 0.05). HOXA5 showed remarkable prognostic value for LUAD patients (P < 0.05). The expression of HOXA4 and HOXA5 in LUAD were negatively correlated with tumor purity and positively correlated with the infiltration of various immune cells such as B cells, T cells and macrophages. HOXA4 and HOXA5 overexpression had notable inhibitory effect on the proliferation, migration and invasion of LUAD cells.

Conclusions: In conclusion, the identified downexpressed HOXA4 and HOXA5 had significant distinguishing ability for LUAD samples and affected the cellular functions of LUAD cells. The low expression of HOXA5 indicated worse overall survival of LUAD patients. Therefore, the two HOXA family genes especially HOXA5 may serve as potential biomarkers for LUAD.

## Introduction

Lung adenocarcinoma (LUAD) is the most common subtype of lung cancer associated with high rates of mortality 1. Due to no obvious symptoms and lack of effective screening tests, most LUAD patients were judged to be in late clinical stage when diagnosed, and missed the window for best opportunity of surgical treatment 1. Although multidisciplinary efforts jointly have greatly promoted the advancement of diagnosis and treatment of LUAD and even target gene therapy have been proposed, the prognosis of LUAD patients remained poor and some patients were tortured by short-term relapse or distant metastasis 2-5. Thus, it was necessary to investigate genes related to the occurrence and progression of LUAD and find new biomarkers for early diagnosis and clinical treatment of LUAD.

HOXA family genes encode proteins containing DNA binding homeobox motifs (HOXA1 to HOXA7, HOXA9, HOXA10, HOXA11, HOXA13). These proteins regulate early embryo segmentation patterns and late developmental events 6. There is literature evidence that the abnormal expression of HOXA family genes including HOXA4 and HOXA5 is closely related to the occurrence and development of diverse human cancers including breast cancer, colorectal cancer and ovarian cancer 7-9. HOXA5 was discovered to present overexpression in breast cancer and served as a prognostic biomarker for colorectal cancer 7-8. The work of Klausen et al. demonstrated the inhibiting effect of HOXA4 on the cell motility and spreading of ovarian cancer cells 9. There have been several studies that reported the expression pattern of HOXA4 and HOXA5 in non-small cell lung cancer as well as the influence of HOXA4 and HOXA5 in biological behaviors of non-small cell lung cancer cells 10-13. Some of our previous work also revealed the influence of aberrant expression of HOXA family genes on the initiation and development of lung cancer. We found that HOXA3 was low expressed in lung adenocarcinoma and had a protective effect on the prognosis of lung cancer patients; whereas HOXA11 and HOXA13 play oncogenic roles in lung adenocarcinoma 14-16. However, no study has been dedicated to the overall appraisal of the clinic-patholoogical significance, impact on cellular biological function and molecular basis of HOXA4 and HOXA5 in LUAD. Herein, we for the first time integrated in-house IHC, RT-qPCR, globally multi-center microarrays and RNA-seq data to comprehensively evaluate the expression and prognostic significance of HOXA4 and HOXA5 in LUAD, and investigated the mechanism of HOXA4 and HOXA5 in the formation and development of LUAD. The functional roles of the two HOXA family genes in LUAD were validated by *in vitro* experiments.

## Methods

### Overview of the expression signatures of HOXA4 and HOXA5 in LUAD and non-cancer lung samples

#### Curation of RNA-seq dataset, microarrays and RT-qPCR results in literature work

LUAD and non-cancerous lung samples in this study were collected from globally multi-center microarray and RNA-seq datasets. We downloaded the fragments per kilobase per million (FPKM) sequencing data of 533 LUAD patients and the accompanying clinical information from GDC portal of TCGA database. Considering the small number of non-cancerous lung cases in TCGA-LUAD project, we downloaded the TPM gene expression matrix of all normal lung specimen in GTEX database as a complement to the non-cancer control of TCGA-LUAD data set. After matching by gene names and removal of batch effect by sva package, the data sets in TCGA and GTEX databases were combined into log2 (TPM + 0.001) standardized gene expression matrix. The relationship between HOXA family gene expression and the clinico-pathological variables of LUAD patients from RNA-seq datasets was analyzed through independent students' t test or analysis of variance (ANOVA), where the expression value of HOXA4 and HOXA5 were expressed as mean (M) ± standard deviation (SD).

All microarrays or RNA-seq datasets containing gene expression value of three or more human LUAD and non-cancerous lung specimens published before 2^nd^, 2021 in GEO, SRA, oncomine and ArrayExpress databases were also included for differential expression analysis. For microarrays eligible for inclusion, we used the limma package of R software (v.3.6.1) to perform inter-group correction, standardization the expression matrix with log2 function, and converting the probe name to official gene names according to the soft formatted family files or array design files. Microarrays from the same platforms were aggregated and processed into a large expression matrix without batch effect by sva package in R software v.3.6.1. Additionally, we also searched in literature databases including Pubmed, CNKI, Wanfang, VIP and sinomed databases to seek studies containing sufficient expression data of HOXA4 and HOXA5 in three or more LUAD and non-cancer lung samples.

### IHC experiments

A total of 100 LUAD and matched paracarcinoma lung tissues were sampled from LUAD patients in The First Affiliated Hospital of Guangxi Medical University. Polyclonal antibody for HOXA4, HOXA5 (ThermoFishier Scientific), broad spectrum secondary antibody (Shanghai Changdao Biotechnology Co., Ltd), Quickblocktm immunostaining primary antibody diluent (Beyotime Biotechnology), 95% ethanol, 75% ethanol and DAB concentrated Kit (Shanghai Changdao Biotechnology Co., Ltd) were the agents used for immunohistochemistry (IHC). Immunostaining of tissues slides of 100 LUAD and 100 normal lung samples was conducted following the procedures of kit instructions. The immunostaining intensity of all IHC images from weak, medium to strong was assigned the scores of 1, 2 and 3. Then, the number of all cells and the percentage of positively stained cells were counted and calculated in five random fields of microscopy. The proportion of positively stained cells were scored as 1, 2 and 3, corresponding to the proportion of positively stained cells reaching less than 25%, between 25% and 75% and more than 75%. The product of staining intensity scores and scores of percentage of positive staining was the final IHC score of each image. When the IHC score is less than four, the IHC results were considered negative. While an IHC score of more than four indicated positive IHC staining. The IHC scores of HOXA4 and HOXA5 between LUAD and non-cancer lung cases were compared via paired students' t test. The current research has been approved by the Ethics Committee of The First Affiliated Hospital of Guangxi Medical University (2015-KY-GUOJI-059) and conforms to the provisions of the Declaration of Helsinki. All patients enrolled for the research gave informed consent.

In order to comprehensively evaluate the differential expression of HOXA4 and HOXA5 in the above microarrays, RNA-seq datasets, literature work and in-house IHC results, we extracted the number of samples (n), mean and standard deviation (SD) of expression values of HOXA4 and HOXA5 in LUAD and non-cancerous lung samples in each dataset. The standardized mean difference (SMD) with 95% confidence interval (CI) were deducted from the sorted extracted data for HOXA4 and HOXA5 with the meta package of R software, which was visualized as a forest plot. We also extracted diagnostic data including true positive, false positive, true negative and false negative of HOXA4 and HOXA5 in all included datasets with the proc and ggplot2 packages of R software (v.3.6.1). The arranged diagnostic matrix was sent as the input for Stata (v.14.0) software, and the summarized receiver's operating characteristic (SROC) curves were drawn for evaluation of the overall discrimination ability of HOXA4 and HOXA5 in discerning LUAD from non-cancer LUAD samples.

### The impact of HOXA4 and HOXA5 on the prognosis of LUAD patients

The online tool KM plotter was employed for appraising the prognostic significance of HOXA4 and HOXA5 on the overall survival or progression-free survival of LUAD patients from multiple probe sets. The different survival conditions of LUAD patients with high or low HOXA4 or HOXA5 expression was presented by Kaplan-Meier survival curves. Splitting of LUAD patients was based on the median expression value of HOXA4 or HOXA5 and Log-rank test plus hazard ratio (HR) values quantified the degree of survival difference.

### Correlations between HOXA4 or HOXA5 and the immune infiltration in LUAD

Correlation analysis of the expression of HOXA4 or HOXA5 and the tumor purity as well as the immune infiltration level in LUAD was conducted through TIMER database (http://cistrome.dfci.harvard.edu/TIMER/), where various immune cells including CD4+ T cells, B cells, neutrophils, CD8+ T cells, macrophages and dendritic cells in LUAD were involved in analysis. The correlation analysis was exhibited as a set of scatter diagrams and P <0.05 indicated statistical significance.

### Correlation between HOXA4 or HOXA5 and the functional state of LUAD cells

We analyzed the associations between HOXA4 or HOXA5 and the functional state of LUAD cells using resources of single cell sequencing available in Cancer SEA database. A total of 14 function state signatures collected from databases such as HCMDB, Cyclebase and StemMapper were stored in Cancer SEA database 17. Spearman's rank correlation analysis was applied for estimating the links between HOXA4 or HOXA5 and the functional state of LUAD cells. Association pairs of gene-state that met the criteria of false discovery rate <0.05 and correlation >0.3 were considered as significant.

### The upstream transcriptional regulatory network of HOXA4 and HOXA5

To investigate the potential upstream transcriptional regulators of HOXA4 and HOXA5, we referred to the NetworkAnalyst (http://www.networkanalyst.ca) database to predict miRNAs or transcription factors (TFs) that possibly modulate the transcription or expression of HOXA4 or HOXA5. The intertwined relationships between predicted miRNAs, TFs, HOXA4 and HOXA5 were exported as topological network.

### The correlations between SIRT1 expression and HOXA4 or HOXA5 expression in LUAD

SIRT1 (SIRT1) is a histone deacetylase that depends on nicotinamide adenine dinucleotide 18. Several studies have pointed out the overexpression and oncogenic effect of SIRT1 in LUAD 18-20. To investigate whether the aberrant expression of HOXA4 and HOXA5 in LUAD were associated with SIRT1, we performed Pearson correlation analysis with Graphpad Prism v. 8.0.1. based on normalized expression data of HOXA4, HOXA5 and SIRT1 in three datasets covering large LUAD samples (TCGA-GTEx, ArrayExpress_Affymetrix and GPL6884).

### Multiscale clustering of geometrical network (MEGENA) analysis for HOXA4 and HOXA5 in LUAD

Differential expression analysis was performed for log2 (TPM + 0.001) standardized TCGA-GTEx gene expression matrix and differentially expressed genes (DEGs) (log2 FC>2 or <-2 and adj.P <0.05) were reserved. Because RNA-seq dataset from TCGA database covered the expression data of both HOXA4 and HOXA5 in large LUAD samples, MEGENA analysis was performed on log2(x+0.001) normalized RNA-seq expression matrix of reserved DEGs, HOXA4 and HOXA5. All steps of MEGENA analysis strictly followed the runscript of MEGENA package in R sotftware v.4.0. (Supplementary file 1). Module genes that co-expressed with HOXA4 and HOXA5 were identified and functionally annotated via ClusterProfiler package in R software v.4.0. False discovery rate and adjusted P value less than 0.05 stated significance of co-expression and enrichment of biological functions or KEGG pathways.

### *In vitro* experiments

#### Cell culture

The human LUAD cell lines A549 and HCC827 were obtained from National Collection of Authenticated Cell Cultures with short tandem repeat (STR) qualification report. The cell line was cultured in Roswell Park Memorial Institute 1640 (RPMI1640) media containing 10% fetal bovine serum (FBS). The cell lines were cultured in the atmosphere of 37℃ and 5% carbon dioxide.

#### Plasmid construction and transfection

Lentivirus systems including LV-HOXA4 and LV-HOXA5 plus negative control virus CON335 were constructed by the Jikai Gene. The A549 and HCC827 cells (1×10^5^) were inoculated in six-well plates and cultured until the confluence of cells reached 20-30%. We used HitransG virus transfection reagent to carry out the transfection according to the manufacturer's instructions. Efficiency of HOXA4 and HOXA5 overexpression was detected by using real time quantitative reverse transcription PCR (RT-qPCR). The primer pairs used for HOXA4 and HOXA5 in RT-qPCR were: 5'-ATAACGGAGGGGAGCCTAAG-3' (HOXA4, forward) and 5'-GCTCAGACAAACAGAGCGTG-3' (HOXA4, reverse); 5'-AACTCATTTTGCGGTCGCTAT-3' (HOXA5, forward) and 5'-TCCCTGAATTGCTCGCTCAC-3' (HOXA5, reverse). The expression value of HOXA4 and HOXA5 expression was quantified using the formula of 2-^ΔΔ^Ct.

The protocols of performing CCK8 proliferation experiments, scratch test and Transwell invasion experiments were described explicitly in previous work 21.

### Statistical analysis

All experiments were performed more than three times. Statistical analyses for *in vitro* experiments were performed using Statistical Package SPSS 22.0. The proliferation, migration and invasion ability of LUAD cells in HOXA4 or HOXA5-overexpression groups and control groups was compared by independent students' t test.

## Results

### Overview of the expression signatures of HOXA4 and HOXA5 in LUAD and non-cancer lung samples

**Figure 1:**
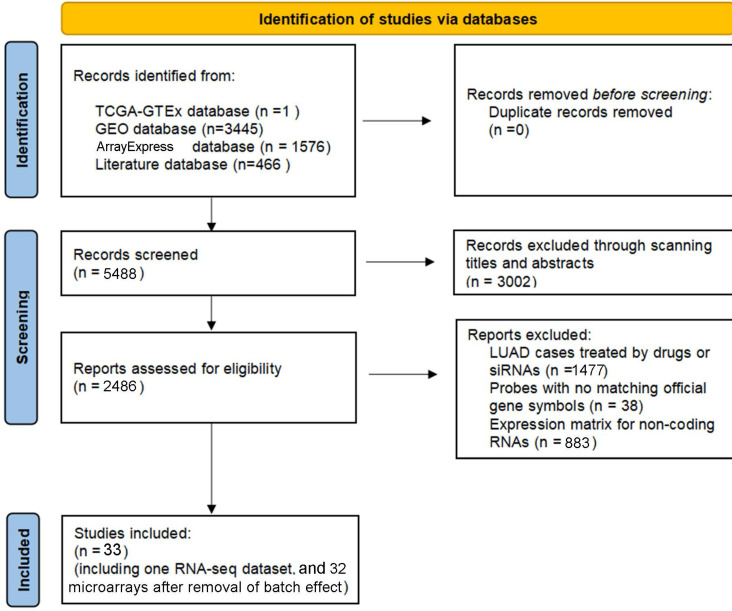
The inclusion process of eligible microarrays and RNA-seq datasets for differential expression analysis.

**Figure 2:**
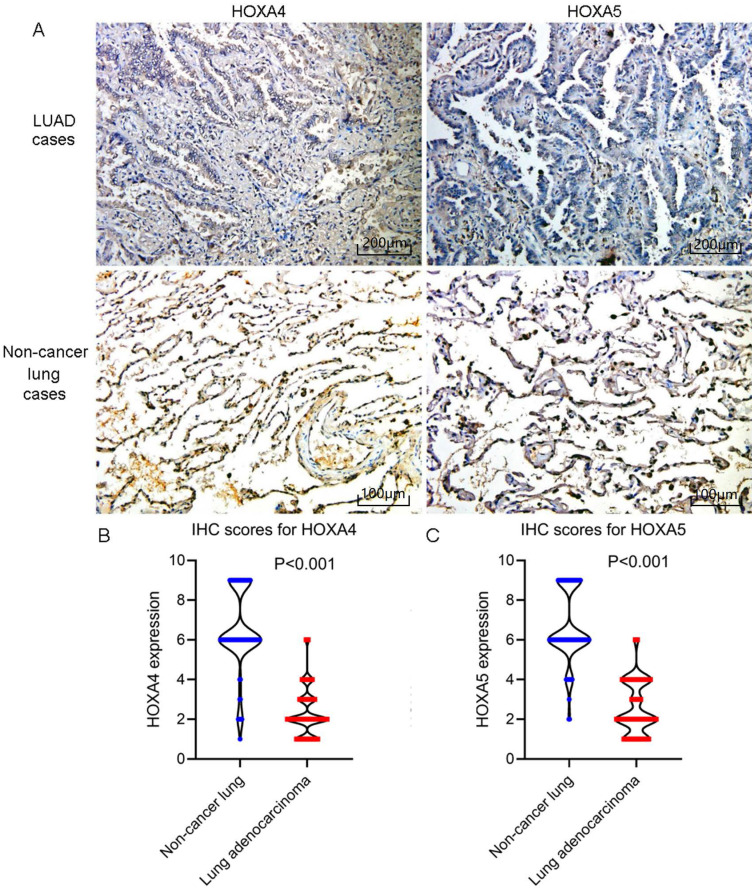
IHC staining results of HOXA4 and HOXA5 in LUAD and non-cancer lung cases. A. Images of IHC staining of HOXA4 and HOXA5 in LUAD and non-cancer lung samples. B. Violin plot of the differential IHC scores of HOXA4 in LUAD and non-cancer lung samples. C. Violin plot of the differential IHC scores of HOXA5 in LUAD and non-cancer lung samples.

**Figure 3:**
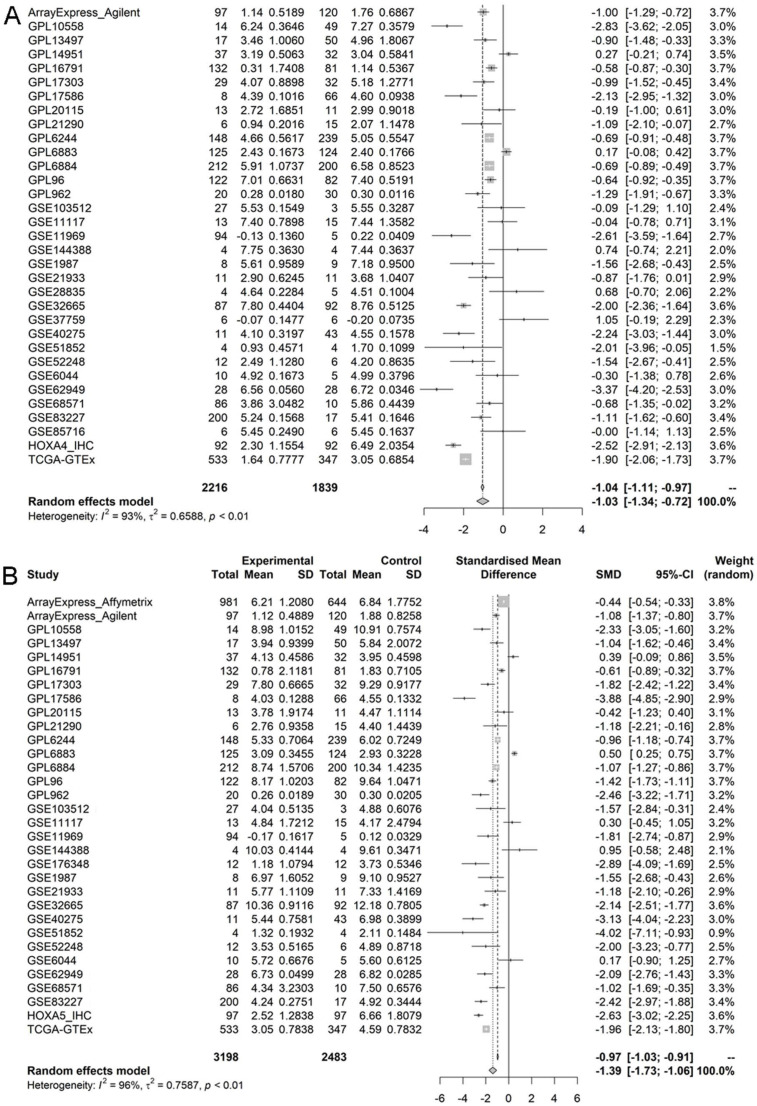
Forest plots of pooled standard mean deviation values for HOXA4 and HOXA5 in LUAD. A. The overall expression data of HOXA4 in all datasets. B. The overall expression data of HOXA5 in all datasets.

**Figure 4:**
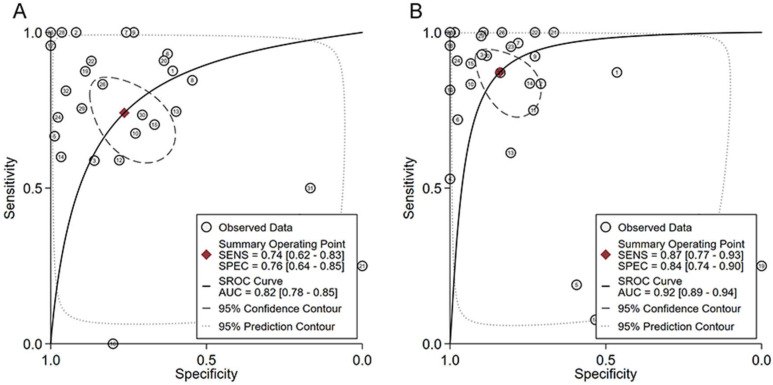
The ability of HOXA4 and HOXA5 in distinguishing LUAD from non-cancer lung samples. A. The summarized receiver's operating characteristics curves for HOXA4 (A) and HOXA5 (B) in all datasets.

**Figure 5:**
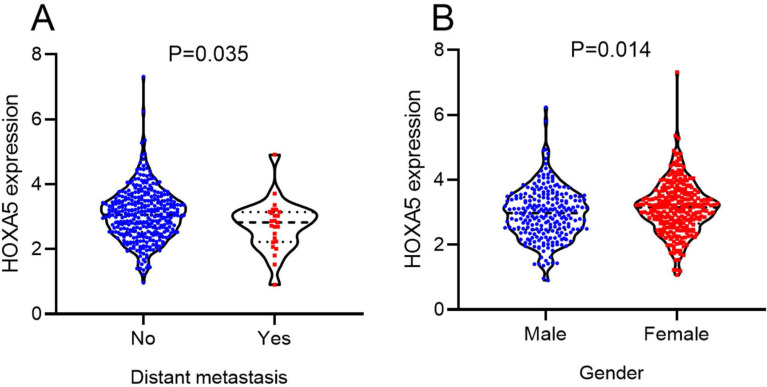
The significant relationships between HOXA5 expression and clinical features of LUAD patients from RNA-seq dataset. Distribution of HOXA5 expression between LUAD patients in different groups of gender and distant metastasis.

According to the selecting criteria, 32 microarrays in total from GEO and Arrayexpress databases were enrolled for the differential expression analysis, the detailed information and selection process of which was summarized in Table S1 and Figure 1. IHC experiments for HOXA4 and HOXA5 confirmed significant down-expression of them in LUAD tissues. While HOXA4 and HOXA5 presented high level of protein expression in nucleus of epithelial cells of normal alveoli and bronchus tissues, negative immunostaining of HOXA4 and HOXA5 protein was observed in the nucleus and cytoplasm of LUAD cells (P<0.001, Figure 2). All LUAD and non-cancer lung samples from in-house IHC experiment, external RNA-seq and microarray datasets amounted to 3201 and 2494, respectively. The forest plots in Figure 3 revealed remarkable down-regulation of HOXA4 and HOXA5 in LUAD. The differential expression of HOXA4 and HOXA5 in LUAD also showed preferable ability of discriminating LUAD from non-cancer lung samples (Figure 4). Clinico-pathological analysis for HOXA4 and HOXA5 in LUAD suggested that LUAD patients without distant metastasis presented higher HOXA5 expression compared to LUAD patients with distant metastasis (P=0.035) (Figure 5). Female LUAD patients exhibited higher HOXA5 expression than male LUAD patients (P=0.014) (Figure 5).

### The impact of HOXA4 and HOXA5 on the prognosis of LUAD patients

**Figure 6:**
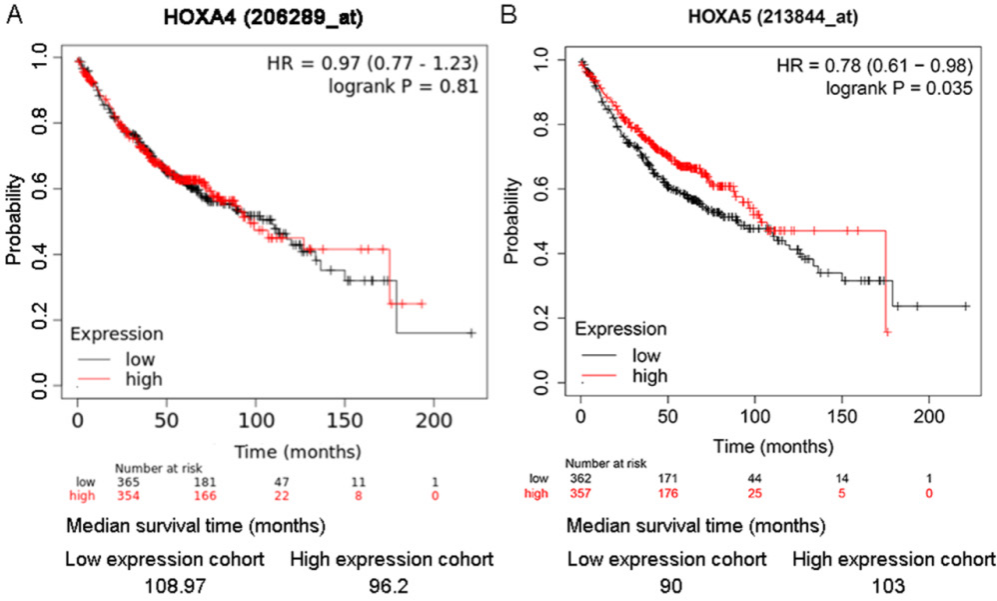
The effect of differential expression of HOXA4 and HOXA5 on the overall survival of LUAD patients in multiple probe sets. A. Kaplan-Meier survival curves for LUAD patients with high or low HOXA4 (A) and HOXA5 (B) expression.

As shown in the Kaplan-Meier survival curves, LUAD patients with higher expression of HOXA5 had greater probability of overall survival than LUAD patients with lower expression of HOXA5 (HR=0.78; P=0.035) (Figure 6). There was no significant difference between the overall survival probability of LUAD patient groups with low HOXA4 expression and high HOXA4 expression (P>0.05, Figure 5).

### Correlations between HOXA4 or HOXA5 and the immune infiltration of LUAD

**Figure 7:**
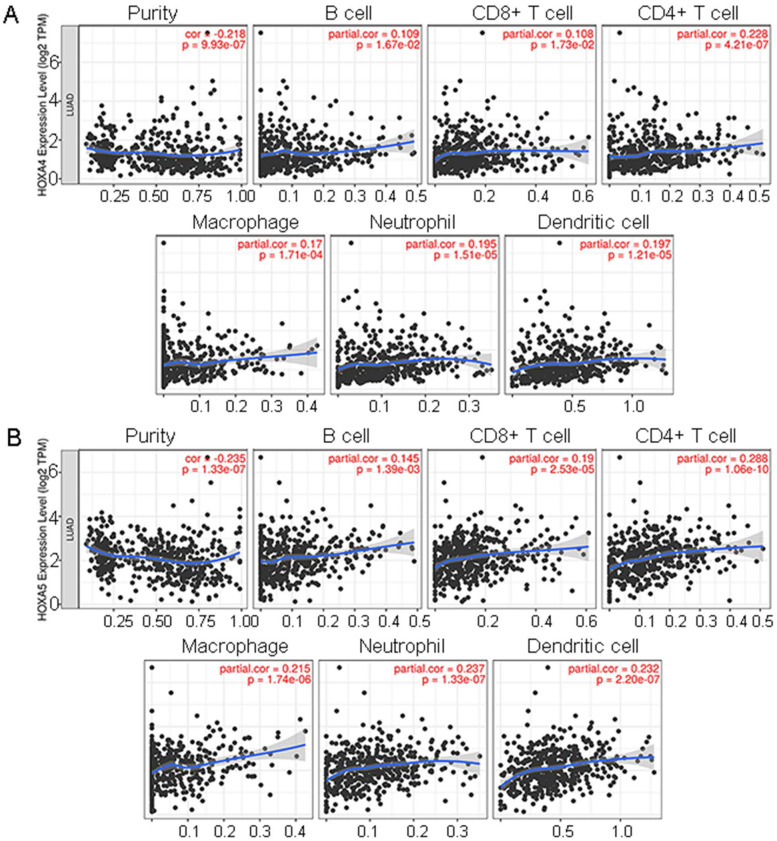
The correlations between expression of HOXA4, HOXA5 and the infiltration level of various immune cells or tumor purity in LUAD. Panels of scatter diagrams were plotted for HOXA4 (A) and HOXA5 (B).

It was disclosed from the correlation diagrams that the infiltration level of B cell, CD8+ T cell, CD4+ T cell, macrophages, neutrophil and dendritic cells were positively linked with the expression of HOXA4 and HOXA5 (P<0.05). Tumor purity was negatively associated with the expression of HOXA4 and HOXA5 in LUAD (Figure 7, P<0.05).

Correlation between HOXA4 or HOXA5 expression and the functional state of LUAD cells

**Figure 8:**
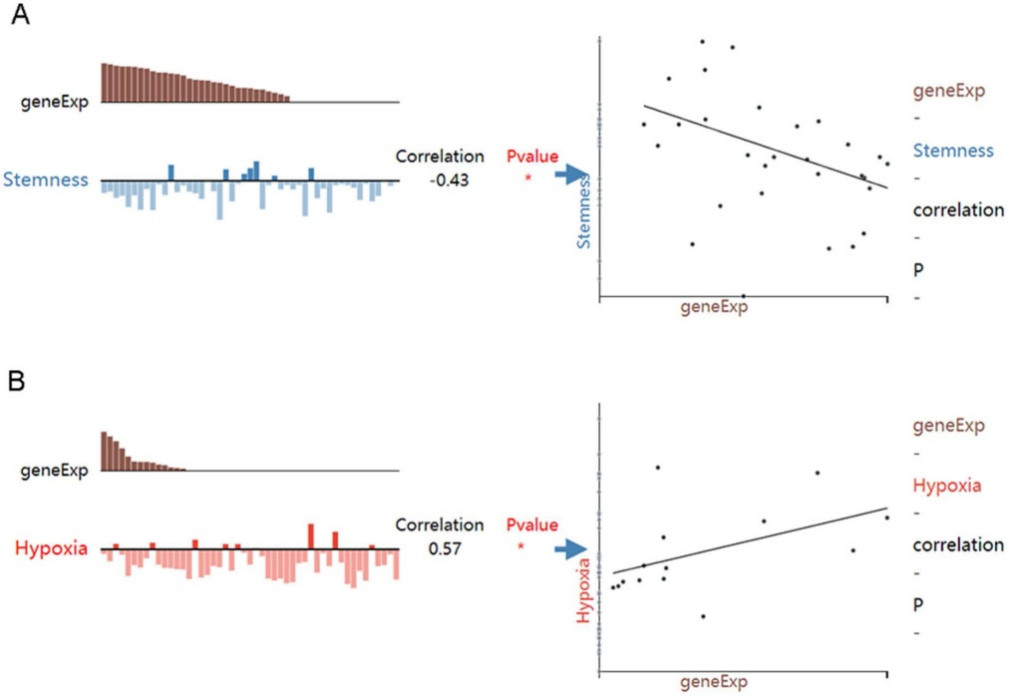
The associations between expression of HOXA4 or HOXA5 and the functional state of LUAD cells. geneExp: gene expression.

While HOXA4 expression was negatively correlated with the stemness of LUAD cells (r=-0.43, P<0.05), HOXA5 expression was positively associated with the hypoxia state of LUAD cells (r=0.57, P<0.05) (Figure 8).

### The upstream transcriptional regulatory network of HOXA4 and HOXA5

**Figure 9:**
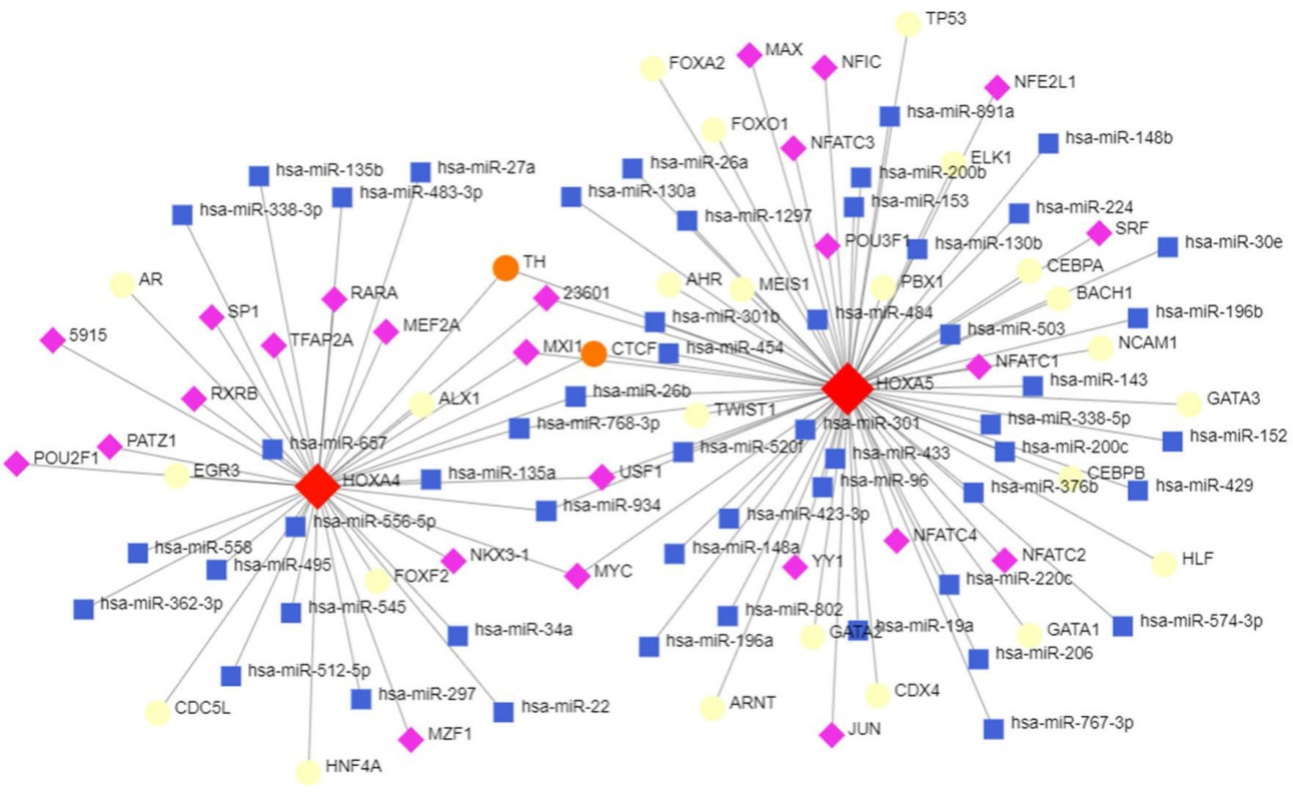
The potential upstream transcriptional regulators of HOXA4 and HOXA5. Transcription factors, miRNAs and HOXA4 or HOXA5 were marked as diamonds, squares and circles in the network.

The topological network in Figure 8 indicated that a series of miRNAs and TFs such as hsa-miR-26b, hsa-miR-96, hsa-miR-30e, USF1, POU2F1 and GATA3 co-regulated the transcription and expression of HOXA4 and HOXA5 (Figure 9).

### The correlations between SIRT1 expression and HOXA4 or HOXA5 expression in LUAD

While HOXA4 expression was positively correlated with SIRT1 expression in GPL6884 dataset (r=0.153, p=0.026), the correlation analysis for TCGA-GTEx dataset revealed contradictory and insignificant results (Figure S1). There was no significant relationship between SIRT1 expression and HOXA5 expression in TCGA-GTEx and ArrayExpress_Affymetrix datasets (p<0.05) (Figure S1).

Multiscale clustering of geometrical network (MEGENA) analysis for HOXA family genes in LUAD

**Figure 10:**
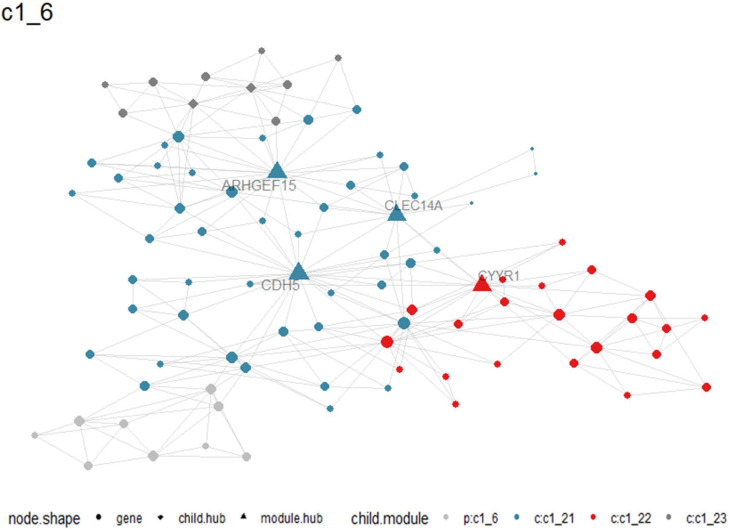
Co-expression between HOXA4 or HOXA5 and significant differantially expressed genes in c1_6 module. Particularly, hub genes in the module were highlighted as triangles.

**Figure 11:**
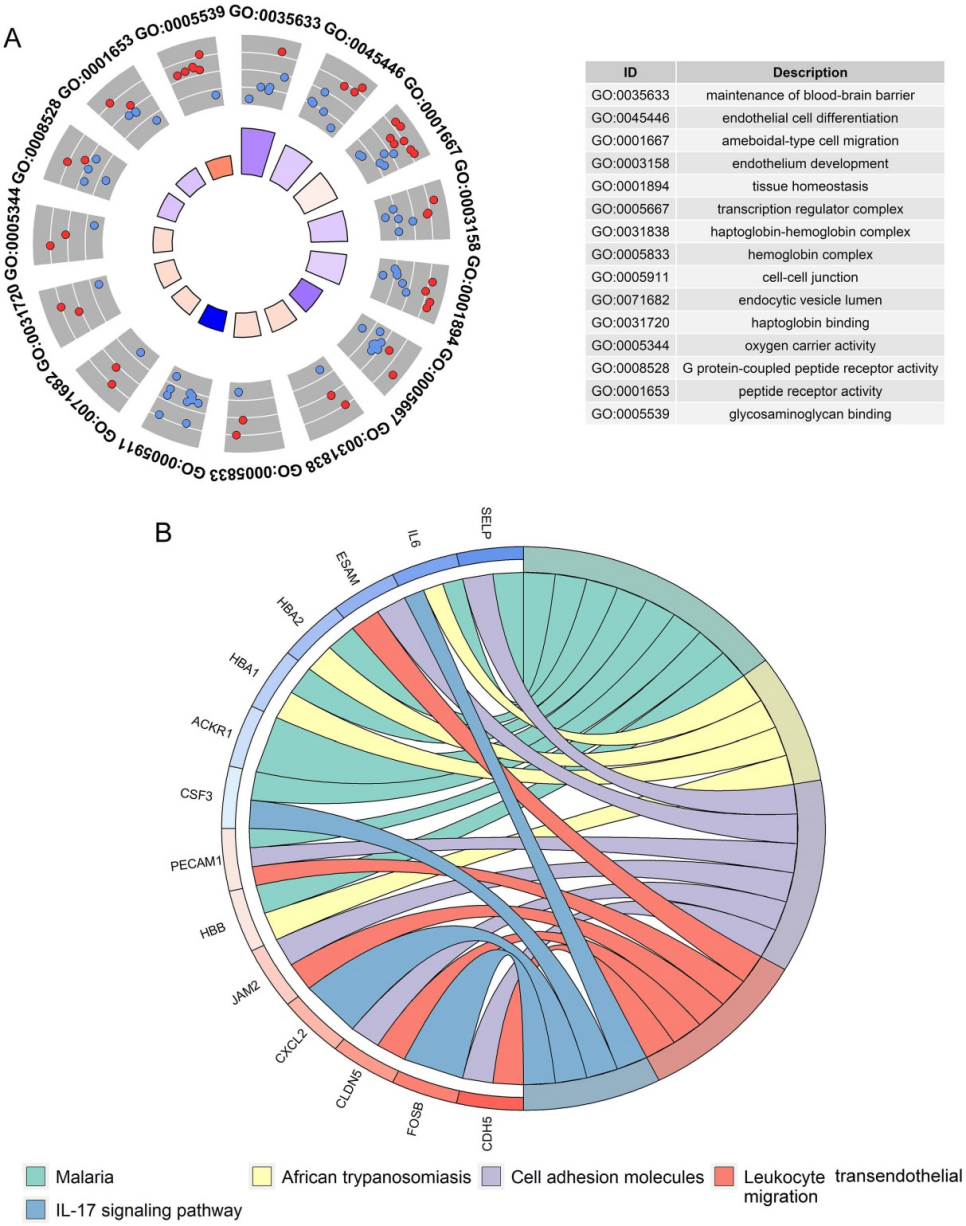
Functional annotations for genes co-expressed with HOXA4 and HOXA5 in LUAD. A. Significant terms of biological process and molecular functions. B. Significant terms of KEGG pathways.

A total of 405 up-regulated DEGs and 201 down-regulated DEGs were reported from differential expression analysis for RNA-seq expression matrix of 533 LUAD samples and 347 non-cancer lung samples in TCGA database (log2FC>2&log2FC<-2, adj.P<0.05). Results from MEGENA analysis based on RNA-seq expression matrix of the 606 DEGs in LUAD revealed dozens of co-expressed modules (Figure S2). Specifically, 88 genes were co-expressed with HOXA family genes in c1_6 module and several genes including ARHGEF15, CDH5, CYYR1 and CLEC14A were designated as hub genes due to high degree of connectivities with other genes (Figure 10). Genes co-expressed with HOXA family genes were significantly clustered in biological processes, molecular functions and KEGG pathways including endothelial cell differentiation, G protein-coupled peptide receptor activity and cell adhesion molecules (Figure 11).

The effect of HOXA4 and HOXA5 on the proliferation, migration and invasion of LUAD cells

Among HOXA family genes with significant aberrant expression and prognostic value in LUAD, we selected HOXA4 and HOXA5 for further cellular function analysis out of research interests. After transfection of the constructed lv-HOXA4 and lv-HOXA5 lentiviruses in A549 and HCC827 cell lines, the PCR results reflected successful lentivirus transfection. The mRNA levels of HOXA4 and HOXA5 in the lv-HOXA4 and lv-HOXA5 lentiviruse groups notably exceeded those in corresponding CON335 and non-treated groups (P < 0.05) (Figure S3).

**Figure 12:**
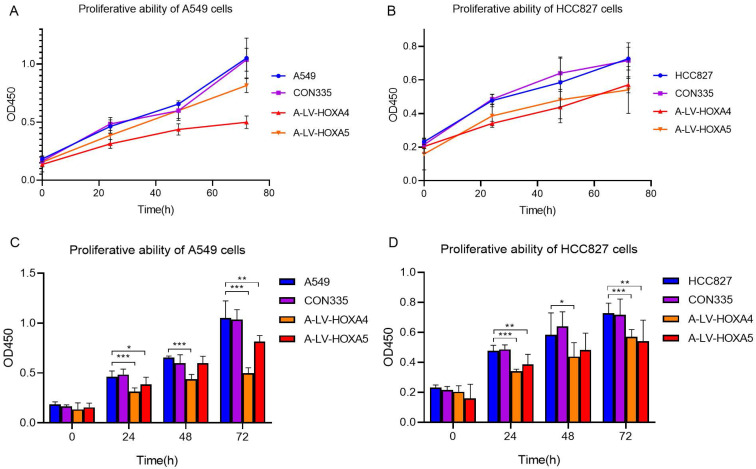
Changes in proliferation ability of A549 cell line and HCC827 cell line after over-expression of HOXA4 and HOXA5. A. Line chart of OD450 values for different groups of A549 cells. B. Line chart of OD450 values for different groups of HCC827 cells. C. Bar chart of OD450 values for different groups of A549 cells. D. Bar chart of OD450 values for different groups of HCC827 cells. *: P<0.05. **: P<0.01. ***: P<0.001.

In order to explore whether HOXA4 and HOXA5 had impact on the proliferation of LUAD cells, CCK-8 experiment was carried out. The results showed that the cell proliferation of A549 cells in lv-HOXA4 and lv-HOXA5 transfected groups was significantly inhibited compared with blank control group (con335) and untreated A549 cells 72 hours after transfection (P < 0.05). Similarly, there was a trend of decrease in cellular proliferation in lv-HOXA4 and lv-HOXA5 transfected HCC827 cells 24 hours after transfection. The suppression of proliferation was most significant at 72h in lv-HOXA4 and lv-HOXA5 transfected HCC827 cells (P<0.05) (Figure 12).

**Figure 13:**
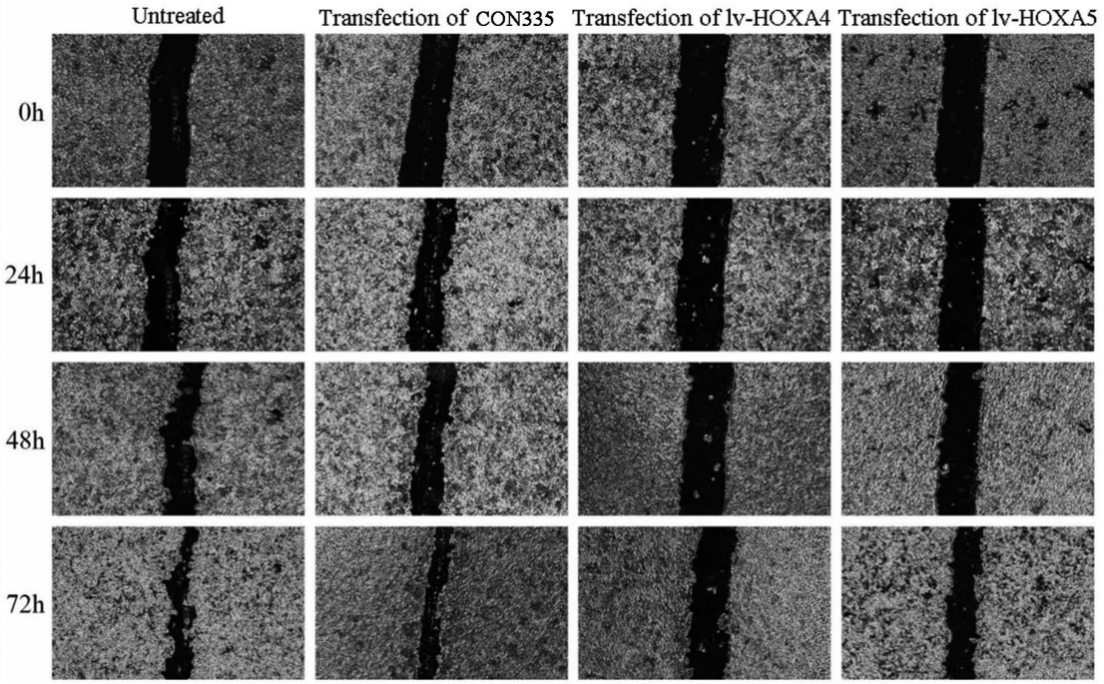
The effect of over-expression of HOXA4 and HOXA5 on the migration ability of A549 cells detected by wound healing test. Photos for scratch and healing areas were taken at 0h, 24, 48h and 72h after transfection with negative control or lv-HOXA4 and lv-HOXA5 lentiviruses.

Scratch assay was subsequently performed for assessing the influence of HOXA4 and HOXA5 overexpression on the migration of LUAD cells. It can be seen from the photos of scratch area that the wound-healing rate of A549 cells transfected with lv-HOXA4 and lv-HOXA5 significantly lagged behind that of the CON335 and non-treated groups at the same time points of 24, 48 and 72h (P<0.05) (Figure 13). Notable inhibition of wound-healing rate was also observed in HCC827 cells transfected with lv-HOXA4 and lv-HOXA5 at 48 and 72h (P<0.05) (Figure S4) (Figure S5).

**Figure 14:**
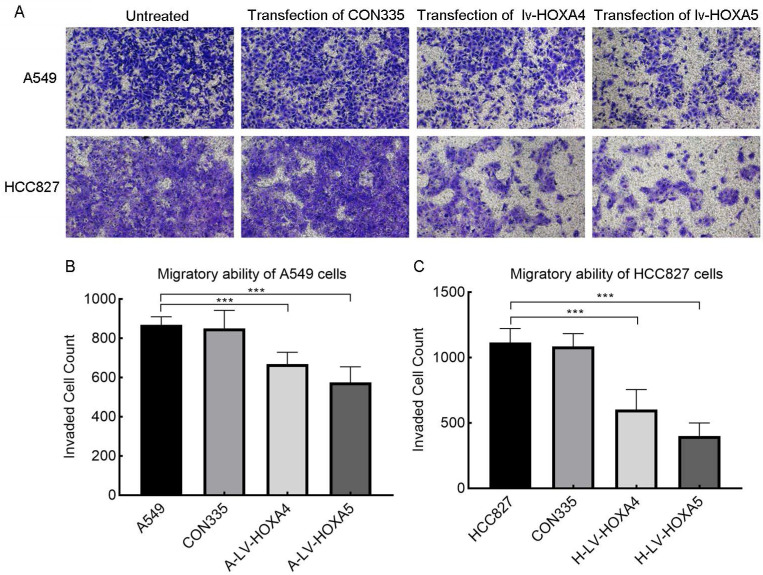
The effect of over-expressed HOXA4 and HOXA5 on the migration ability of A549 and HCC827 cells verified by Transwell migration assay. A. Images of migration of A549 and HCC827 cells in different treatment groups. B. Bar chart of invaded cell count in different treatment groups of A549 cells. C. Bar chart of invaded cell count in different treatment groups of HCC827 cells. *: P<0.05. **: P<0.01. ***: P<0.001.

In addition, we also verified the effect of HOXA4 and HOXA5 overexpression on the migrative ability of LUAD cells by Transwell chamber migration experiment. It was found that the number of migrating A549 and HCC827 cells in the lv-HOXA4 and lv-HOXA5 transfected groups was significantly reduced compared with the CON335 and non-treated groups (Figure 14), which implied suppressed migration of LUAD cells (P < 0.05).

## Discussion

The role of HOXA4 and HOXA5 in the occurrence and progression of a variety of human cancers has been scatteredly reported in previous literature studies 7, 8, 22, 23. However, there is no systematic study on the differential expression, prognostic significance and potential molecular mechanism of HOXA4 and HOXA5 in LUAD.

The most attractive highlight of the current study was comprehensively appraising the differential expression and clinico-pathological significance of HOXA4 and HOXA5 in LUAD through integrating in-house IHC experiment results, all existing microarrays and RNA sequencing data from diverse cohorts. Decreased expression of HOXA4 and HOXA5 in LUAD was discovered in the present work. The research of Abe m and other scholars reported that only the expression of HOXA5 and HOXA10 in LUAD was obviously higher than that in non-cancerous lung specimen 24. Although the conclusions of the present work differed from the conclusions of Abe m's work, the large sample pool of 3201 LUAD and 2494 non-cancerous lung samples collected from various cohorts worldwide made the results of the present study more convincing. The further clinico-pathological analysis results reflected the roles of HOXA5 in promoting or restraining the clinical development of LUAD. Wang et al reported that HOXA5 could suppress the metastasis of LUAD cells by regulating cytoskeletal remodeling 25. This study provides a possible explanation for the negative relationships between HOXA5 down-expression and lymph node metastasis of LUAD patients. The latest clinical diagnosis and treatment guidelines for lung cancer issued by the Chinese Medical Association recommended that examination of EGFR, ALK, ROS1 were indispensable for NSCLC detection and other genes including BRAF, MET, HER2, KRAS and RET should also be considered for NSCLC detection 26. In this study, the correlations between HOXA4 or HOXA5 expression and mutations of the above genes in LUAD patients were not statistically significant (the results were not shown). We conjectured that the abnormal expression of HOXA4 or HOXA5 and the above events of genetic mutations might exert influences on different aspects of the initiation and progression of LUAD. We further explored the influence of HOXA4 and HOXA5 expression on the prognosis of LUAD patients for the first time. The consistency in trends of differential expression and prognostic value proved the clinical potential of HOXA5 in the risk stratification of LUAD patients.

It was necessary to investigate the molecular basis of HOXA4 and HOXA5 in LUAD before the clinical application of them; thus, we delved into the mechanism of HOXA4 and HOXA5 in the oncogenesis of LUAD. Positive correlations between HOXA4 or HOXA5 and infiltration level of various immune cells were revealed by immune correlation analysis, which echoed with the findings in several prior literature studies. Heidi et al. uncovered the frequent dysregulation of HOXA family genes including HOXA2, HOXA3, HOXA4, HOXA7, HOXA9, HOXA10 and HOXA11 in multiple myeloma, a disease featured by malignant growth of plasma cells in the bone marrow 27. The close relationships between down-regulated HOXA4 and HOXA5 and B cells found in the present study could be traced back from the crucial roles of HOXA family genes in the growth of immature B-cell fractions and early lymphoid progenitors 28. Based on the above results and evidence, we speculated that HOXA4 or HOXA5 may participate in the remodeling of tumor micro-environment to influence the occurrence and development of LUAD. With respect to the relationships between functional states of LUAD cells and HOXA4 or HOXA5 expression, significant gene-state pairs of HOXA4-stemness and HOXA5-hypoxia suggested that HOXA4 and HOXA5 might block the development of LUAD through decreasing stemness of LUAD cells or increasing the hypoxia level of tumor microenvironment. The upstream transcriptional regulation of HOXA4 and HOXA5 was equally deserving of attention and we explored the possible transcriptional regulation mechanisms of HOXA4 and HOXA5 in LUAD through predicting miRNAs and TFs. Most of the predicted miRNAs and TFs except for hsa-miR-301b have not been verified to target HOXA4 or HOXA5 by previous researchers 29. The newly found miRNA/TF-HOXA4/HOXA5 network in this study might shed light on the abnormal regulation mechanisms behind the down-expression of HOXA4 and HOXA5 in LUAD. The role of epigenetic regulators in tumor targeted therapy has been of critical interest 30. The anti-aging gene sirtuin 1 (SIRT1) has been reported to be associated with LUAD and non-small cell lung cancer and high protein level of SIRT1 has been shown to indicate poor prognosis of lung cancer patients 18, 31-33. SIRT1 is involved with transcription dysregulation including deacetylation of transcription factors such as p53 and DNA methylation 34-36. Prior researchers showed that HOXA5 and p53 co-worked to retard lung cancer cell invasion and served as good prognostic factors in non-small cell lung cancer 12. It is assumed that the transcriptional regulation of HOXA5 might be involved in the critical deacetylaton of p53 by SIRT1 in LUAD and non-small cell lung cancer, and that the marked down-regulation of HOXA4 and HOXA5 in LUAD may be linked to transcriptional dysregulation of SIRT1 which is now strongly linked to LUAD and non-small lung cancer. Thus, we investigated the correlations between SIRT1 expression and HOXA4 or HOXA5 expression in LUAD. However, the results from this study were not strong enough to support the potential regulation of SIRT1 on the transcription of HOXA4 or HOXA5 in LUAD. Then, we extended the mechanism study of HOXA4 and HOXA5 in LUAD to the co-expressed genes and analyzed the functional enrichment of gene co-expressed with HOXA4 or HOXA5 in LUAD. Among the significant biological process or molecular function terms clustered by HOXA4 and HOXA5-related genes, some were closely correlated with cell growth, proliferation, apoptosis and migration. There have been literature documents of the involvements of HOXA4 and HOXA5 in formation and progression of various human cancers through active or inhibitory roles in proliferation, growth, migration and apoptosis in cancer cells 37-42. Analysis results from this part implied possible functional roles of HOXA4 and HOXA5 in LUAD. Because biological process and molecular function terms linked to cell proliferation and migration appeared with high frequency in the list, we carried out *in vitro* experiment to validate the impact of HOXA4 and HOXA5 on the proliferation, migration and invasion of LUAD cells. The results from *in vitro* experiments corroborated the analysis results from functional annotation and indicated that HOXA4 and HOXA5 might retard the development of LUAD via suppressing the proliferation, migration and invasion of LUAD cells.

There were also some limitations of the current work. The immune correlations of HOXA4 or HOXA5 expression in LUAD and the interactions between HOXA4 or HOXA5 and predicted miRNAs, TFs or co-expressed genes should be checked by further experiments.

## Conclusions

In conclusion, we demonstrated the remarkable down-regulation of HOXA4 and HOXA5 in LUAD. HOXA5 is expected to be a clinical prognostic marker for LUAD patients. HOXA4 or HOXA5 may serve as tumor-suppressors in the occurrence and development of LUAD by acting on immune cells or affecting the proliferation, migration and invasion of LUAD cells.

## Supplementary Material

Supplementary figures.

Supplementary table.

## Abbreviations

LUAD: lung adenocarcinoma
IHC: immunohistochemistry
FPKM: fragments per kilobase per million
ANOVA: analysis of variance
SD: standard deviation
SMD: standardized mean difference
SROC: summarized receiver's operating characteristic
HR: hazard ratio
TF: transcription factor
MEGENA: multiscale clustering of geometrical network analysis
DEG: differentially expressed genes
RT-qPCR: real time quantitative reverse transcription PCR
