# Supplementary material for: Expression Landscape and Functional Roles of HOXA4 and HOXA5 in Lung Adenocarcinoma
Source: Int J Med Sci. 2022 Mar 6;19(3):572–87. doi: 10.7150/ijms.70445 (PMC8964330; doi:10.7150/ijms.70445)
Supplement: Supplementary file 1 — Supplementary figures. [file ijmsv19p0572s1.pdf]

Supplementary materials

Table S1. Basic elements of included datasets for differential expression analysis.

Figure S1. Correlations between HOXA4 or HOXA5 expression and SIRT1 expression in LUAD. A: Scatter plot of HOXA4-SIRT1 expression pair in TCGA-GTEx dataset; B: Scatter plot of HOXA4-SIRT1 expression pair in GPL6884 dataset; C: Scatter plot of HOXA5-SIRT1 expression pair in TCGA-GTEx dataset; D: Scatter plot of HOXA5-SIRT1 expression pair in ArrayExpress_Affymetrix dataset.

Figure S2. The co-expressed modules clustered by HOXA4, HOXA5 and all significant differentially expressed genes from RNA-seq dataset of LUAD. The node graph displayed the hierarchy of the co-expressed modules.

Figure S3. The comparisons of expression of HOXA4 and HOXA5 in LUAD cell lines after transfection with negative control or lv-HOXA4 and lv-HOXA5 lentiviruses. A: Bar graph of expression values for A549 cell line; B: Bar graph of expression values for HCC827 cell line. *: P<0.05.

Figure S4. The effect of over-expression of HOXA4 and HOXA5 on the migration ability of HCC827 cells detected by wound healing test. Photos for scratch and healing areas were taken at 0h, 24, 48h and 72h after transfection with negative control or lv-HOXA4 and lv-HOXA5 lentiviruses.

Figure S5. The scratch healing ability of A549 and HCC827 cell lines after over-expression of HOXA4 and HOXA5. A. Line chart of scratch healing area values for different groups of A549 cells. B. Line chart of scratch healing area values for different groups of HCC827 cells. C. Bar chart of scratch healing area values for different groups of A549 cells. D. Bar chart of scratch healing area values for different groups of HCC827 cells. *: P<0.05. **: P<0.01. ***: P<0.001.

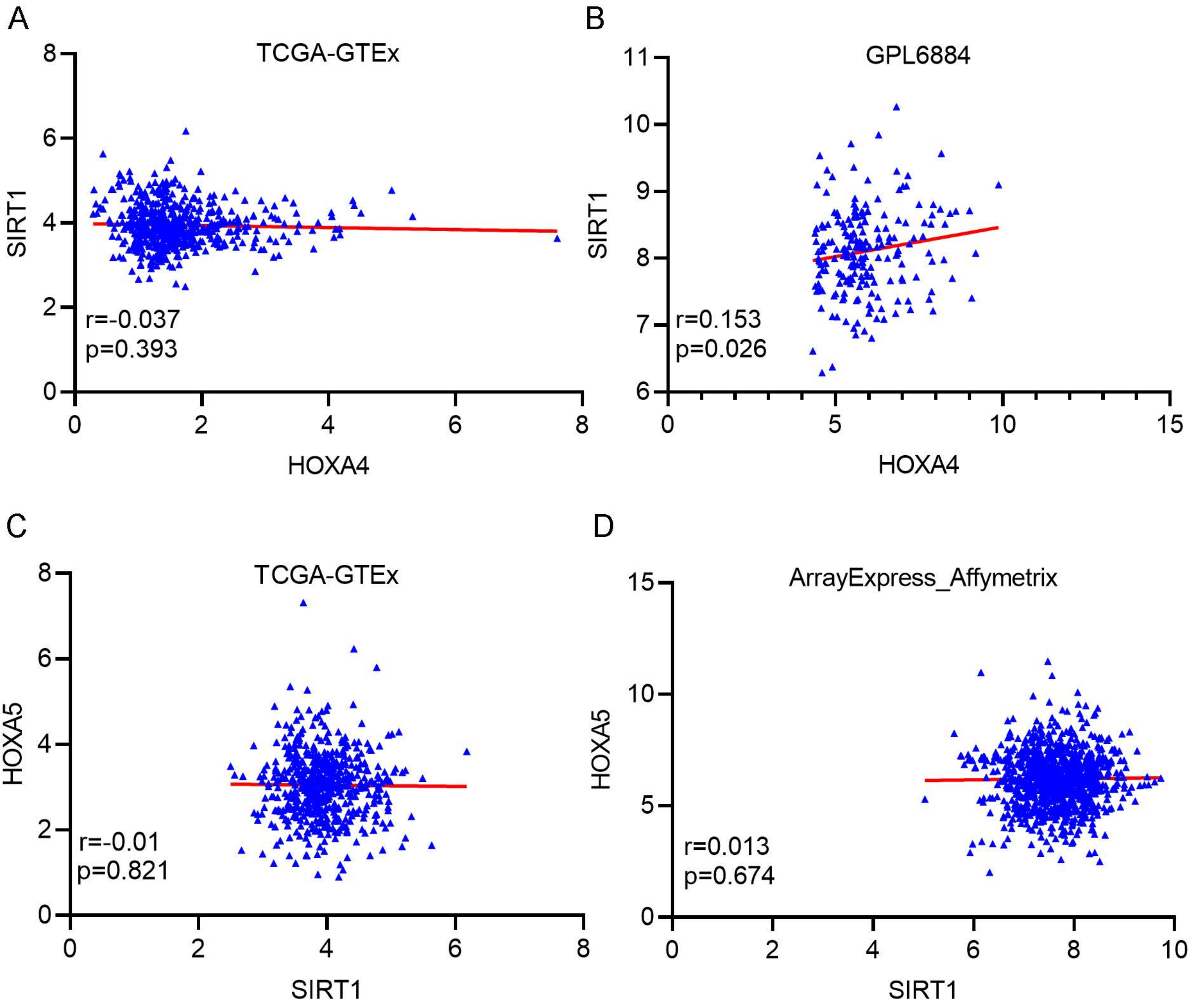
A
TCGA-GTEx
SIRT1
HOXA4
r=-0.037
p=0.393
B
GPL6884
SIRT1
HOXA4
r=0.153
p=0.026
C
TCGA-GTEx
HOXA5
SIRT1
r=-0.01
p=0.821
D
ArrayExpress_Affymetrix
HOXA5
SIRT1
r=0.013
p=0.674

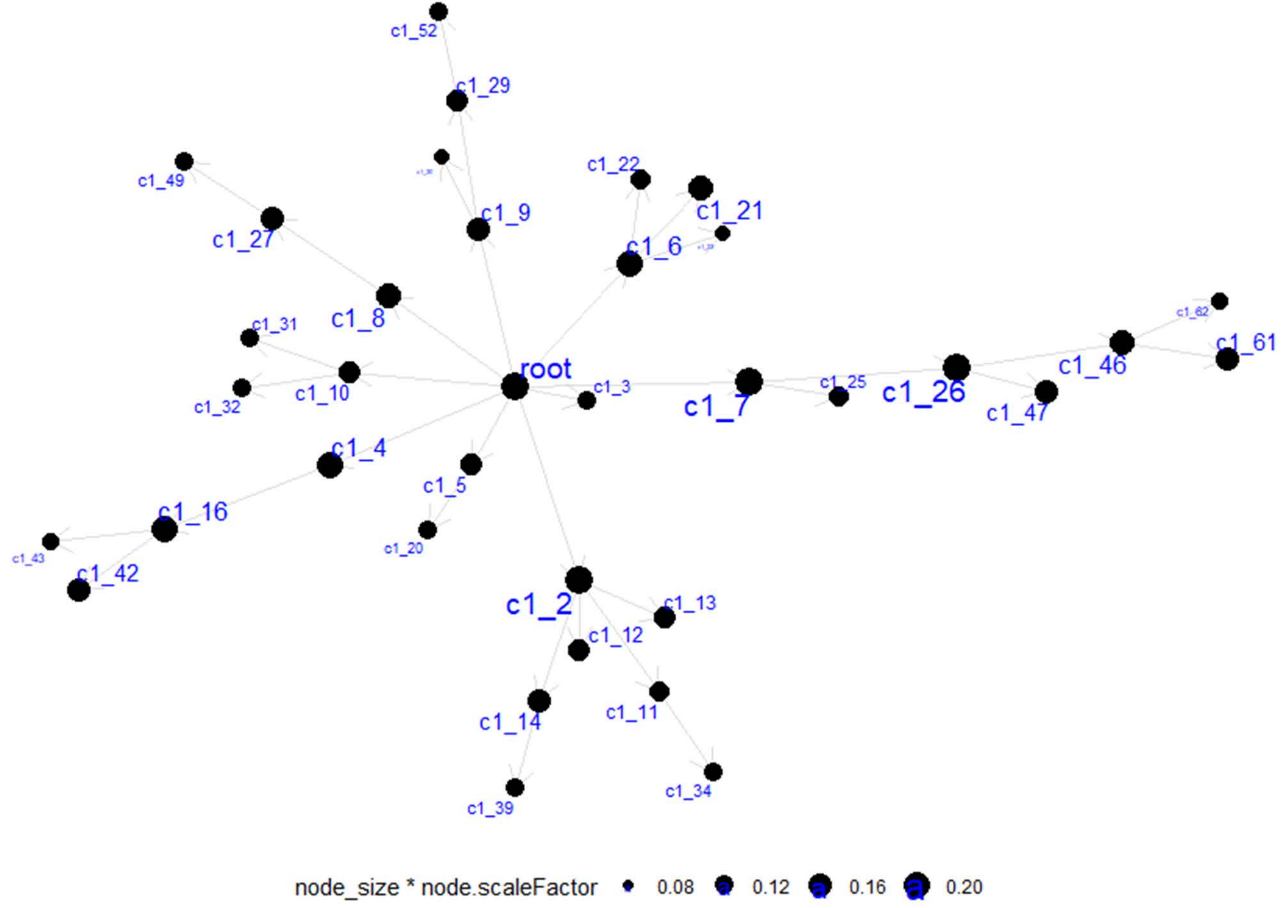

c1_52
c1_29
c1_49
c1_9
c1_22
c1_21
c1_27
c1_6
c1_8
c1_62
c1_31
c1_61
root
c1_46
c1_25
c1_3
c1_10
c1_26
c1_32
c1_7
c1_47
c1_4
c1_5
c1_16
c1_20
c1_43
c1_42
c1_2
c1_13
c1_12
c1_14
c1_11
c1_39
c1_34
node_size * node.scaleFactor
0.08
0.12
0.16
0.20

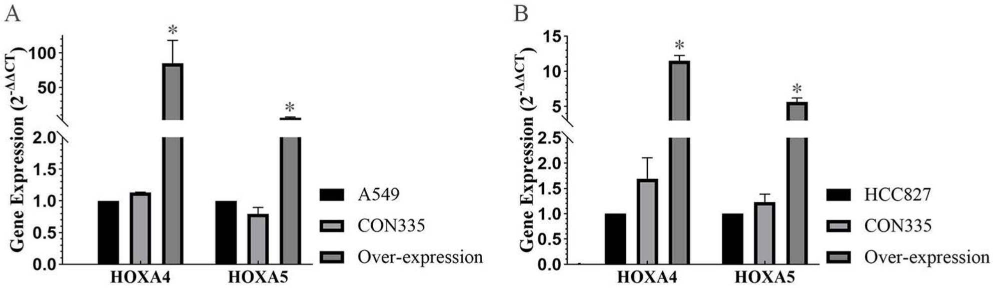
A
B
Gene Expression (2^-ΔΔCT)
100
50
2.0
1.5
1.0
0.5
0.0
15
10
5
2.5
*
HOXA4
HOXA5
A549
HCC827
CON335
Over-expression

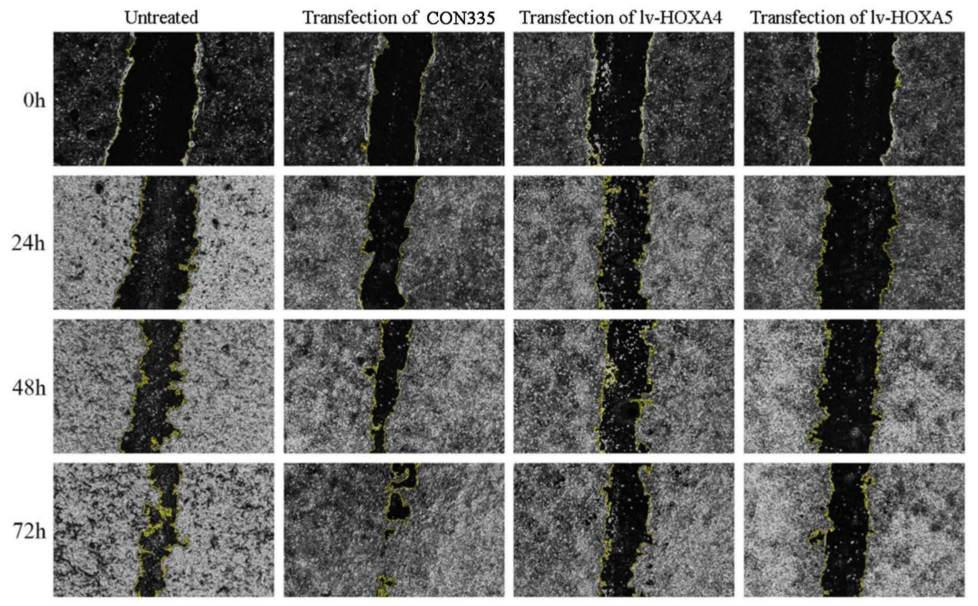
Untreated
Transfection of CON335
Transfection of lv-HOXA4
Transfection of lv-HOXA5
0h
24h
48h
72h

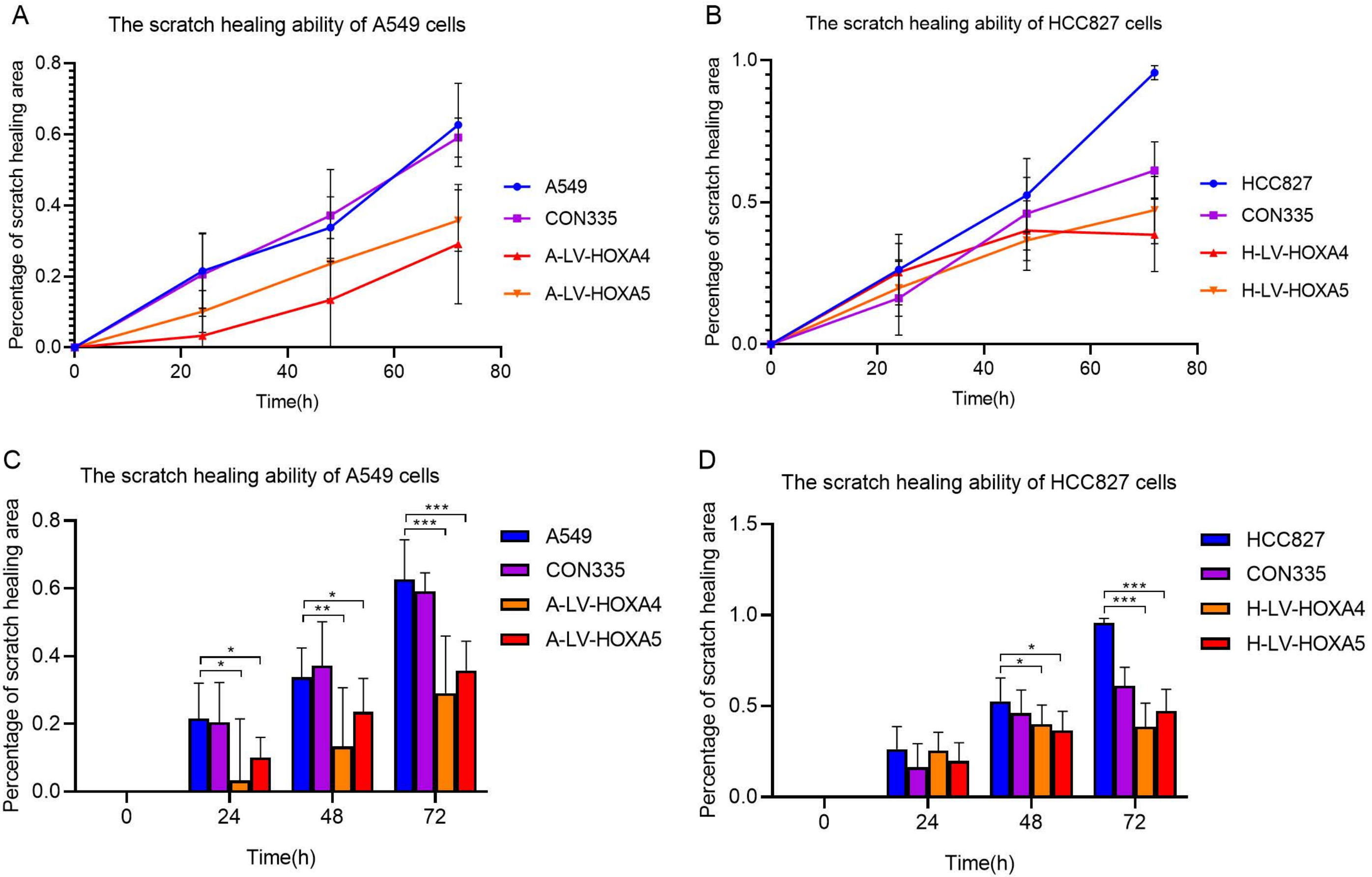

A
The scratch healing ability of A549 cells
Percentage of scratch healing area
Time(h)
A549
CON335
A-LV-HOXA4
A-LV-HOXA5
B
The scratch healing ability of HCC827 cells
Percentage of scratch healing area
Time(h)
HCC827
CON335
H-LV-HOXA4
H-LV-HOXA5
C
The scratch healing ability of A549 cells
Percentage of scratch healing area
Time(h)
A549
CON335
A-LV-HOXA4
A-LV-HOXA5
D
The scratch healing ability of HCC827 cells
Percentage of scratch healing area
Time(h)
HCC827
CON335
H-LV-HOXA4
H-LV-HOXA5
